# The long‐term effect of dupilumab on chronic hand eczema in patients with moderate to severe atopic dermatitis—52 week results from the Dutch BioDay Registry

**DOI:** 10.1111/cod.14104

**Published:** 2022-04-24

**Authors:** Angelique N. Voorberg, Geertruida L. E. Romeijn, Marjolein S. de Bruin‐Weller, Marie L. A. Schuttelaar

**Affiliations:** ^1^ Department of Dermatology, University of Groningen University Medical Center Groningen Groningen The Netherlands; ^2^ Department of Dermatology and Allergology University Medical Center Utrecht Utrecht The Netherlands

**Keywords:** atopic dermatitis, biological, dupilumab, hand eczema, quality of life, treatment

## Abstract

**Background:**

The hands are a common predilection site of atopic dermatitis (AD). Dupilumab is licensed for the treatment of AD but not for chronic hand eczema (CHE), while CHE is challenging to treat.

**Objectives:**

To evaluate the long‐term effect of dupilumab on hand eczema (HE) in patients with AD from the BioDay Registry.

**Methods:**

A prospective observational study of adult patients with HE, treated for AD with dupilumab. Patients with a HE severity of at least moderate at baseline were considered for analysis. Patients with other concomitantly systemic immunosuppressive treatments were excluded. Clinical effectiveness on HE severity, using the Hand Eczema Severity Index (HECSI) and photographic guide, and health‐related quality of life, using the Quality of Life in Hand Eczema Questionnaire (QOLHEQ), were evaluated.

**Results:**

A total of 72 patients were included. HECSI‐75 was achieved by 54/62 patients (87.1%) and HECSI‐90 by 39/72 (62.9%) at 52 weeks. Based on the photographic guide, 56/62 patients (90.3%) achieved the endpoint of ‘clear’ or ‘almost clear’. Mean QOLHEQ reduction was −63.5% (95% confidence interval −38.23 to −27.41). There was no difference in response between HE subtypes.

**Conclusions:**

The results from this study hold promise for dupilumab to be a suitable treatment option for isolated CHE.

## Graphical abstract

Dupilumab for atopic hand eczema.

- Dupilumab is licensed for the treatment of atopic dermatitis (AD) but not for chronic hand eczema (CHE), while CHE is challenging to treat.
- Dupilumab gave long‐term reduction of hand eczema severity and long‐term improvement of hand eczema‐related quality of life in patients with AD and concomitant moderate to very severe hand eczema.
- The results from this study hold promise for dupilumab to be a suitable treatment option for isolated CHE.

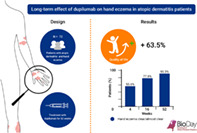

## 1 INTRODUCTION

The hands are a common predilection site of atopic dermatitis (AD), with a prevalence of hand eczema (HE) in patients with AD up to an odds ratio of 4.06 (95% confidence interval [CI] 2.72‐6.06) as found in a meta‐analysis.^1^ In patients with AD who have chronic HE (CHE), the wrists and the dorsal side of the hands are most commonly affected,^2^ but patients with AD can also have vesicular HE or hyperkeratotic HE.

While mild HE can generally be treated using emollients, combined with topical corticosteroids or topical calcineurin inhibitors, the treatment for moderate to severe HE remains challenging. Systemic treatment options are scarce, because alitretinoin is the only approved treatment for all types of HE while it is primarily effective in hyperkeratotic HE.^3^ Other off‐label, secondary treatment options, such as cyclosporine, may be effective, but long‐term treatment can lead to serious adverse events including hypertension, nephrotoxicity, and risk of malignancy.^4^, ^5^

Dupilumab is a human monoclonal antibody, binding to the interleukin (IL)‐4 receptor α chain, inhibiting IL‐4 and IL‐13, both type 2 inflammatory cytokines that mediate the pathogenesis of AD. Several retrospective studies and case series have been published on the efficacy of dupilumab in patients with HE.^6^, ^7^ In a previous publication, we published the effect of dupilumab on HE in patients with AD up until 16 weeks.^8^ In this study, we evaluated the long‐term (52 weeks) effect of dupilumab on HE in patients with AD.

## 2 METHODS

### 2.1 Study design

This study included patients from the Dutch BioDay Registry at the Department of Dermatology from the University Medical Center Groningen. The BioDay Registry is a prospective observational cohort study in which patients with moderate to severe AD are enrolled who receive novel systemic therapies for their AD in daily practice.^9^ This study is a follow‐up study to the previous publication in which we published the effect of dupilumab on HE in patients with AD up until 16 weeks,^8^ including the same patients from the BioDay Registry. The Medical Ethical Review Board of the University Medical Center Groningen (Groningen, the Netherlands) confirmed that the current study did not fall under the scope of the Medical Research Involving Human Subjects Act, and approved of its study protocol (reference: METc 2018/344). All patients provided written informed consent before inclusion.

### 2.2 Study population

The study population consisted of adult patients with moderate to severe AD (≥18 years) with concomitant HE, who received treatment with dupilumab subcutaneously (600 mg loading dose, followed by 300 mg every 2 weeks). Patients were included between October 2017 and June 2021. Inclusion criteria consisted of a diagnosis of AD according to the UK Working Party criteria,^10^ a diagnosis of HE according to the current guidelines,^11^, ^12^ and a minimum HE severity of ‘moderate’ on the photographic guide by Coenraads et al.^13^ at baseline. All patients who used systemic immunosuppressive or immunomodulating drugs during the study period were excluded from the data analyses. A minimum washout period of 4 weeks before baseline was applied for immunosuppressive and immunomodulating drugs, with an exception for cyclosporine (minimum washout period of 2 weeks) and prednisolone (minimum washout period of 1 week). Usage of emollients, topical corticosteroids, and topical calcineurin inhibitors, as well as the usage of inhalation, nasal, and ocular steroids was permitted. Lastly, patients with known relevant contact sensitizations, without avoidance of these allergens, were excluded.

### 2.3 Outcome measures

The general course of disease severity over 52 weeks is graphically presented as the mean (percentage) change of the Hand Eczema Severity Index (HECSI). The HECSI is an instrument used to rate the severity of six efflorescences of HE (erythema, induration/papules, vesicles, fissures, scaling, and oedema) and the extent of the lesions on five distinct areas of the hand by using standard scales.^14^ The score ranges from 0 to 360, with higher scores representing more severe disease. Improvement was defined as a minimum improvement after 52 weeks on the HECSI of 50% (HECSI‐50), 75% (HECSI‐75), and 90% (HECSI‐90). Furthermore, response to treatment was defined as the achievement of ‘clear’ or ‘almost clear’, and the more strict achievement of ‘clear’ or ‘almost clear’ plus a minimum of two or more steps improvement on the photographic guide compared with baseline.^13^ The photographic guide is a validated 5‐point scale instrument, assessing clear, almost clear, moderate, severe, and very severe based on a set of photographs. For assessing health‐related quality of life (HRQoL) in patients with HE, the Quality of Life in Hand Eczema Questionnaire (QOLHEQ) was used. The QOLHEQ consists out of 30 items that can be summarized according to four domains of HRQoL: impairments because of (1) symptoms, (2) emotions, (3) limitations in functioning, or (4) treatment and prevention. The total QOLHEQ score ranges between 0 and 117, with higher scores indicative of a poor HE‐specific HRQoL.

Besides sociodemographic variables, the following variables were collected at baseline: smoking pack‐years, duration of HE, atopic comorbidities, Investigator Global Assessment for AD severity, occupation (including high risk of developing HE wet work),^15^ irritant contact dermatitis (ICD), patch testing, clinical subtype of HE, and use of previous systemic immunosuppressive/immunomodulatory medication.

### 2.4 Statistical analysis

All continuous outcome measures in the intention‐to‐treat population were analysed using a mixed‐effect model with repeated measures. HECSI values are presented as both the mean percentage change with 95% CIs at the various time points compared with baseline, and the percentage of patients reaching a minimum of 50%, 75%, and 90% improvement on their HECSI scores (HECSI‐50, HECSI‐75, and HECSI‐90) at the various time points (4, 16, 28, 40, and 52 weeks) compared with baseline. QOLHEQ values are presented as the mean percentage change with 95% CIs at the various time points compared with baseline for both the QOLHEQ total scores and the QOLHEQ subscale scores. Patients with missing QOLHEQ data at baseline were excluded from the analysis. Other missing QOLHEQ data, which were all missing ‘completely at random’, were imputed using multiple imputation. Fisher exact test and the independent Student *t*‐test were used to compare percentages and means in independent groups, respectively. Calculations were performed with IBM SPSS Statistics for Windows, version 23.0 (IBM Corp.). A *P*‐value of <.05 was regarded as statistically significant.

## 3 RESULTS

### 3.1 Study population

In total, 72 patients were included in this study. Of these 72 patients, 48 (66.7%) were male. The mean age of the study population was 45.2 years (standard deviation [SD] 13.0). Only two clinical subtypes of HE were observed in our population: chronic fissured HE and recurrent vesicular HE. The majority (72.2%) of patients had a chronic fissured HE. Patient demographics and characteristics at baseline are presented in Table 1.

**TABLE 1:** Baseline demographics and disease characteristics

Characteristic	n = 72
Age, mean (SD)	45.2 (13.0)
Sex, n (%)	
Male	48 (66.7)
Female	24 (33.3)
BMI, median (IQR)	25.6 (22.8‐28.7)
Smoking	
Current smokers, n (%)	24 (33.3)
Ex‐smokers, n (%)	14 (19.4)
Pack‐years, median (IQR)	2.0 (0.0‐16.0)
Duration of disease in years, mean (SD)	26.9 (18.4)
Clinical subtype of HE, n (%)	
Chronic fissured	52 (72.2)
Recurrent vesicular	20 (27.8)
Aetiological factors for HE	
Patch testing performed, n (%)	52 (72.2)
At least one positive reaction to the European baseline series,^22^, ^23^ n (% of n tested)^a^	28 (38.9)
Metals	11 (15.3)
Preservatives	5 (6.9)
Fragrances	8 (11.1)
Rubbers	6 (8.3)
Dyes/colours	4 (5.6)
Topicals	8 (11.1)
Corticosteroids	0 (0.0)
Other	8 (11.1)
Irritant contact dermatitis, n (%)	16 (22.2)
Performing wet work, n (%)	6 (8.3)^b^
Protein contact dermatitis, n (%)	2 (2.8)
Working in a high‐risk occupation for HE, n (%)	24 (33.3)^b^
Baseline HECSI score, median (IQR)	42.0 (20.0‐79.8)
Baseline severity photographic guide, n (%)	
Moderate	40 (55.6)
Severe	24 (33.3)
Very severe	8 (11.1)
Baseline QOLHEQ score, median (IQR)	53.0 (40.0‐75.0)
Baseline QOLHEQ score, subdomain ‘Symptoms’, mean (SD)	16.4 (4.9)
Baseline QOLHEQ score, subdomain ‘Treatment/prevention’, mean (SD)	11.3 (6.1)
Baseline QOLHEQ score, subdomain ‘Emotions’, mean (SD)	14.4 (7.6)
Baseline QOLHEQ score, subdomain ‘Functioning’, median (IQR)	16.0 (7.0‐21.0)
Atopy, n (% of n tested)^c^	58 (80.6)
Asthma	44 (61.1)
Allergic rhinitis	51 (70.8)
Allergic conjunctivitis	40 (55.6)
Total IgE level elevated (≥116 kU/L)^d^	56 (77.8)
Age of onset (AD), median (IQR)	16.5 (4.0‐27.8)
Early onset (0‐2 y), n (%)	42 (58.3)
Childhood onset (3‐11 y), n (%)	17 (23.6)
Adolescent onset (12‐17 y), n (%)	2 (2.8)
Adult onset (18‐50 y), n (%)	9 (12.5)
Late onset (>50 y), n (%)	2 (2.8)
AD localizations, n (%)	
Head/neck	59 (81.9)
Trunk	56 (77.8)
Upper extremities	72 (100.0)
Lower extremities	63 (87.5)
Baseline EASI score, mean (IQR)	22.2 (12.6‐32.6)
Baseline IGA score, n (%)	
Almost clear	1 (1.4)
Mild	9 (12.5)
Moderate	19 (26.4)
Severe	29 (40.3)
Very severe	14 (19.4)
Number of systemic therapies, median (IQR)^e^	2 (1.0‐3.0)
Cyclosporine, n (%)	67 (93.1)
Prednisolone, n (%)	59 (81.9)
Methotrexate, n (%)	26 (36.1)
Azathioprine, n (%)	19 (26.4)
Alitretinoin, n (%)	10 (13.9)
Mycophenolic acid n (%)	5 (6.9)
Mycophenolate mofetil, n (%)	5 (6.9)
Tacrolimus (oral), n (%)	2 (2.8)
Other, n (%)	4 (5.6)

*Note*: Missing values: packyears, n = 3.

Abbreviations: AD, atopic dermatitis; BMI, body mass index; EASI, Eczema Area and Severity Index; HE, hand eczema; HECSI, Hand Eczema Severity Index; IGA, Investigator Global Assessment (for atopic dermatitis); IgE, immunoglobulin E; IQR, interquartile range; QOLHEQ, Quality of Life in Hand Eczema Questionnaire; SD, standard deviation.

^a^ For 38/52 patients, patch testing results were known/reliable (eg, angry back).

^b^ 42/72 patients performed paid work at baseline.

^c^ Based on specific IgE inhalant allergens >0.99; test performed in 69/72 patients.

^d^ Missing in seven patients.

^e^ Number of systemic therapies minus prednisolone.

### 3.2 Safety and drop‐outs

Mild conjunctivitis was the most common adverse event, and was reported in 20 patients (27.8%). In three patients, severe conjunctivitis with limbitis was reported. Blood eosinophilia (>0.40 × 10^9^/L) was also commonly seen among the patients. At baseline (Table 1), 33 out of 72 patients (45.8%) had blood eosinophilia. This proportion increased significantly at 16 weeks, to 61.1%. At 52 weeks, the proportion of patients with blood eosinophilia decreased to 41.7% (30 out of 62 patients).

Of the 72 included patients, 62 patients completed 52 weeks of treatment with dupilumab. Among the patients who stopped treatment with dupilumab, four patients dropped out because of side effects, including severe conjunctivitis with limbitis (n = 3) and the occurrence of multiple verrucae filiformes (n = 1). Four patients dropped out because of ineffectiveness. Of the remaining two patients, one patient was lost to follow‐up and the other patient stopped treatment with dupilumab on patient's own initiative.

In three out of the four patients who dropped out after 28 weeks due to ineffectiveness, improvement of HE was observed compared with baseline (HECSI at drop‐out improved with 25.0%, 54.6%, and 100%, respectively). Both AD and HE symptoms in the remaining patients got worse (Eczema Area and Severity Index [EASI] and HECSI deteriorated with 167.0% and 10.3%, respectively, compared with baseline).

### 3.3 Effectiveness

**FIGURE 1:**
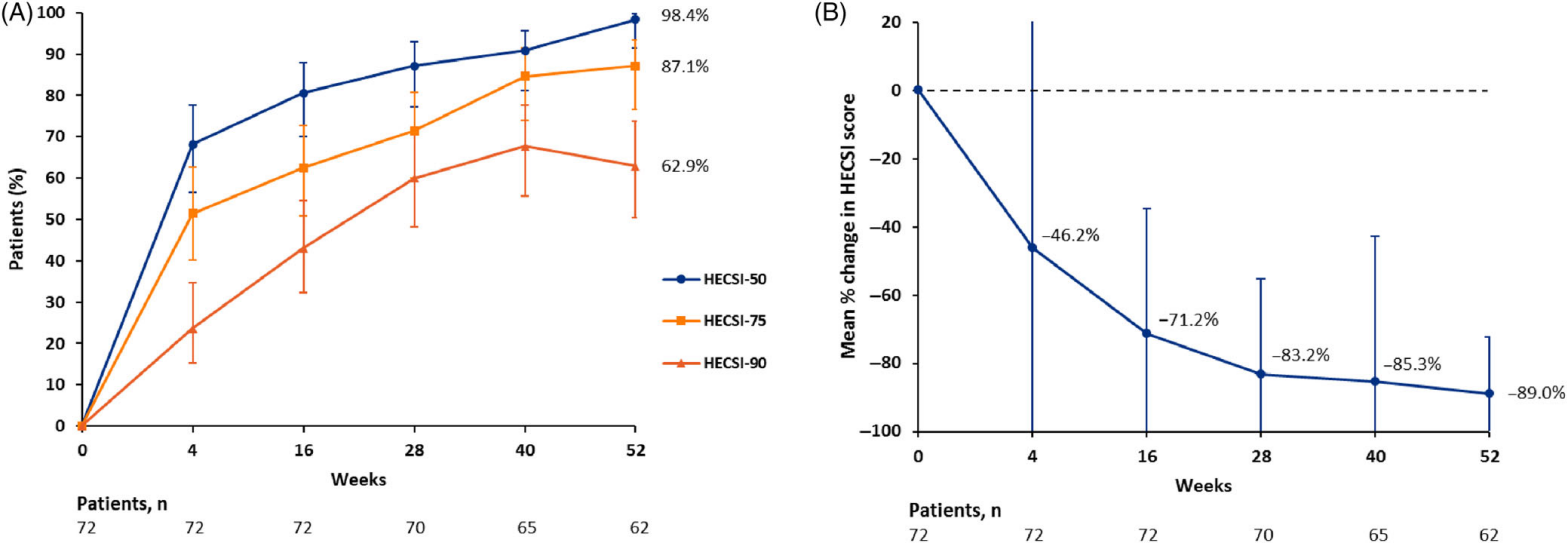
Hand Eczema Severity Index (HESCI) score development during dupilumab treatment in the intention‐to‐treat population. The error bars reflect the 95% confidence intervals. (**A**) Percentages of patients achieving 50%, 75%, and 90% reduction in HECSI score (HECSI‐50, HECSI‐75, and HECSI‐90) from baseline up to 52 weeks. (**B**) Mean percentage change in HECSI score from baseline up to 52 weeks. Negative values indicate improvement

HECSI‐75 was met in 54/62 patients (87.1%) at 52 weeks (Figure 1A). Furthermore, 39 patients (62.9%) achieved HECSI‐90. The mean percentage change of the HECSI at 52 weeks compared with baseline was –89.0% (95% CI −93.1 to −84.5.5; Figure 3).

The proportion of patients achieving HECSI‐75 did not significantly differ between morphological subtypes; in patients with chronic fissured HE, 38 of the 45 (84.4%) achieved HECSI‐75 at 52 weeks, compared with 16 of the 17 patients (94.1%) with a recurrent vesicular HE subtype (*P* = .43). The proportion of patients reaching HECSI‐75 also did not differ between patients with or without concomitant ICD, which was 84.6% and 87.8%, respectively (*P* = .67).

**FIGURE 2:**
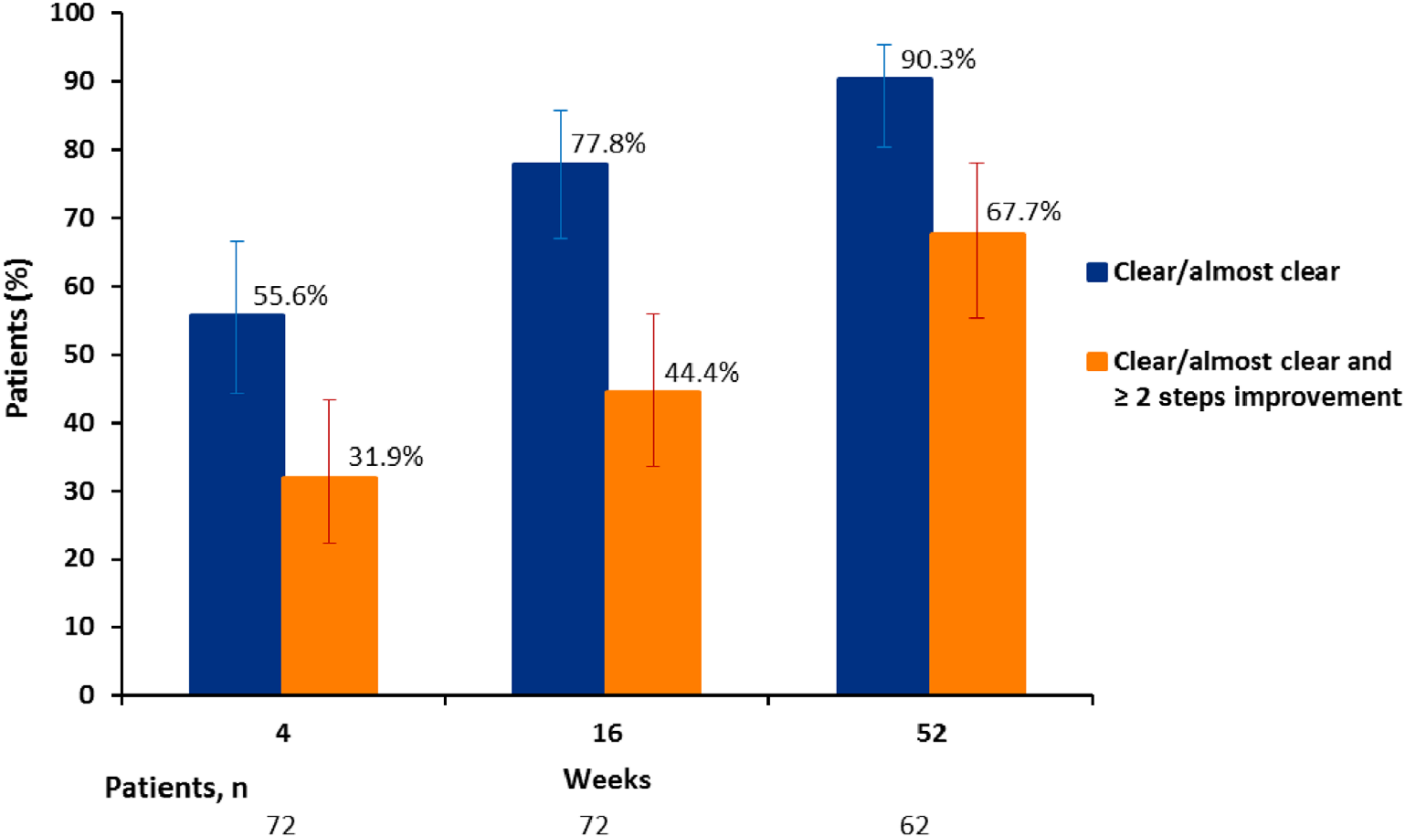
Treatment response based on the photographic guide in the intention‐to‐treat population. The blue bars represent the proportion of patients achieving ‘clear’ or ‘almost clear’. The orange bars represent the proportion of patients achieving both ‘clear’ or ‘almost clear’ and an improvement of at least two steps on the photographic guide. The errors bars reflect the 95% confidence intervals

Based on the photographic guide, 56/62 patients (90.3%) achieved ‘clear’ or ‘almost clear’ at the 52 weeks assessment. Furthermore, 42/62 patients (67.7%) achieved ‘clear’ or ‘almost clear’ and at least two steps improvement on the photographic guide at 52 weeks (Figure 2).

### 3.4 Quality of life

**FIGURE 3:**
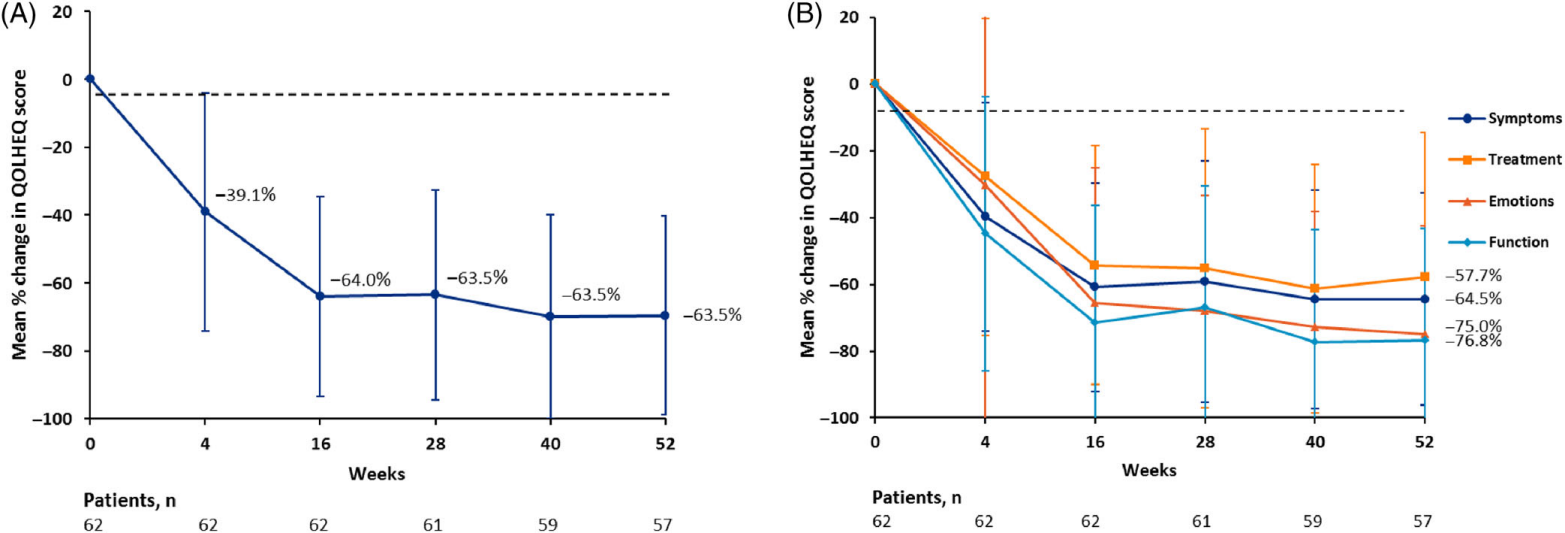
Quality of Life in Hand Eczema Questionnaire (QOLHEQ) score development during dupilumab treatment in the intention‐to‐treat population. Negative values indicate improvement. The error bars reflect the 95% confidence intervals. (A) Mean percentage change in QOLHEQ score from baseline up to 52 weeks. (B) Mean percentage change in QOLHEQ score per subscale from baseline up to 52 weeks

After 52 weeks, a mean decrease of 63.5% (95% CI −71.1 to −55.9) or of 38.1 points (SD 23.3) was observed for the QOLHEQ. The mean total score of the QOLHEQ at 52 weeks was 18.2 points (SD 20.3). A total of 44/57 patients (77.2%) achieved the MIC of 22 points' reduction at 52 weeks.^16^ After 4 weeks, the mean QOLHEQ score was already significantly decreased compared with the baseline score (*P* < .001). The QOLHEQ percentage change for all time points is presented in Figure 3A.

At 52 weeks, a mean decrease of 64.5% (95% CI −72.7 to −56.2) was observed for the domain ‘Symptoms’. For the domain ‘Emotions’ a mean decrease of –75.0% (95% CI −83.4 to −66.6) was found. For the domain ‘Functioning’ a mean decrease of –77.1% (95% CI −85.5 to −68.7) was observed. Lastly, the least mean decrease was found for the domain ‘Treatment and prevention’: −59.7% (95% CI −70.1 to −49.4%). When comparing the four subscales, the mean percentage change at 52 weeks differed significantly between each subscale (*P* < .05). Percentage change per subscale for all time points is graphically shown in Figure 3B.

## 4 DISCUSSION

In this prospective, observational study, we presented data on the long‐term effect of dupilumab on HE in patients with AD. All patients had continuous improvement in HE severity and HE‐specific quality of life after 16 weeks up to 52 weeks. No difference in severity outcomes was found between subtypes of HE.

Compared with our previous publication of the effect of dupilumab on HE in patients with AD after 16 weeks, a higher proportion of patients achieved HECSI‐75 (87.1% after 52 weeks vs 60.0% after 16 weeks). For the photographic guide, we also observed a higher proportion of patients achieving ‘clear’ or ‘almost clear’ after 52 weeks (90.3% vs 76.6% after 16 weeks) at the 52 weeks' assessment.^8^ This indicates even further improvement of HE and a sustained positive effect on HE if patients continue dupilumab treatment after 16 weeks.

In this study in which patients had moderate to very severe HE, a mean moderate impairment in HE‐specific HRQoL was found at baseline. This finding is in line with a recent questionnaire‐based study using the QOLHEQ to study HE‐specific HRQoL among patients with vesicular HE.^17^ A marked improvement in HRQoL was found in more than three‐quarter of the patients, based on reaching the MIC. When specifically looking at the subdomains, the least improvement could be found in the subdomain ‘Treatment and prevention’. This can be explained by the fact that patients still need to avoid irritants and relevant contact allergens, and the use of topical ointments and creams remain necessary for adequate management of HE. The most improvement was found for the subdomain ‘Functioning’, which includes the impact of HE on patients' job, homework, hobbies, washing, dressing, social contacts, and relationship with their family and partner.

The results of this study show that dupilumab might be an effective treatment for CHE in patients with AD. This positive effect on HE has also been shown in several case reports in isolated, nonatopic HE, including vesicular HE, hyperkeratotic HE, allergic contact dermatitis, and ICD.^7^, ^18^ Furthermore, in a recently published study, in which we analysed the transcriptome of vesicular HE through RNA‐sequencing, it was found that *IL4R* was also highly upregulated in lesional HE skin compared with healthy control skin.^19^ This suggests that the IL‐4/IL‐13 pathway might also be involved in isolated HE. These overlaps in pathways between the AD transcriptome and HE transcriptome, and results from previously published case reports and case series in isolated HE, hold promise for dupilumab to be a suitable treatment for moderate to severe CHE in the future.

The main limitation in this study is the use of concomitant topical corticosteroids, up to class IV steroids including clobetasol ointment. This might have affected the observed effect of dupilumab. Furthermore, allergic factors might have influenced the severity score, because 14 of the 42 patients that performed paid work at baseline were working in a high‐risk occupation for HE. In multiple patients (n = 20), patch testing could not be performed due to the severity of their AD. It is not known whether these patients had any (relevant) contact sensitizations.

In conclusion, this study showed that dupilumab might be an effective treatment for moderate to severe CHE in patients with AD, with long‐term clinical effectiveness and great improvement of HE‐specific HRQoL. The efficacy of dupilumab on isolated HE is currently being investigated in phase 2, placebo‐controlled clinical trials.^20^, ^21^ Meanwhile, the results from this study, combined with those from other case studies in different subtypes of HE, hold promise for dupilumab to be a suitable treatment option for isolated, CHE.

## CONFLICTS OF INTEREST

Dr M.L.A.S. is an advisor, consultant, speaker, and/or investigator for AbbVie, Pfizer, LEO Pharma, Regeneron, Sanofi Genzyme, Eli Lilly, and Galderma. She has received grants from Regeneron, Sanofi Genzyme, Novartis, and Pfizer. M.S.B‐W. is a principal investigator, consultant, and advisory board member for AbbVie, Regeneron Pharmaceuticals, Inc, Sanofi Genzyme, Leo Pharma, and Pfizer; and is an advisory board member and consultant for Arena, ASLAN, Galderma, and Eli Lilly. A.N.V. and G.L.E.R. have no conflicts of interest to declare.

## MEDICAL RESEARCH ETHICS COMMITTEE APPROVAL

This study did not fall under the scope of the Medical Research Involving Human Subjects Act, which was confirmed by the local Medical Ethical Review Board of the University Medical Center Groningen (METc 2018/344).

## AUTHOR CONTRIBUTIONS

**Angelique N. Voorberg:** Conceptualization (equal); data curation (equal); formal analysis (lead); investigation (equal); methodology (equal); project administration (equal); resources (equal); validation (equal); visualization (equal); writing – original draft (lead). **Geertruida L. E. Romeijn:** Data curation (equal); investigation (equal); project administration (equal); resources (equal); validation (equal); visualization (equal); writing – review and editing (supporting). **Marjolein S. de Bruin‐Weller:** Data curation (equal); funding acquisition (equal); investigation (equal); methodology (equal); project administration (equal); resources (equal); supervision (supporting); validation (equal); visualization (equal); writing – review and editing (supporting). **Marie L. A. Schuttelaar:** Conceptualization (equal); data curation (equal); funding acquisition (equal); investigation (equal); methodology (equal); project administration (equal); resources (equal); supervision (lead); validation (equal); visualization (equal); writing – original draft (supporting); writing – review and editing (lead).

## Data Availability

The data that support the findings of this study are available from the corresponding author upon reasonable request.
